# Serum ErbB2 concentration positively correlated to the glycemic variations in newly diagnosed Type 2 diabetic patients

**DOI:** 10.1038/s41598-022-07549-x

**Published:** 2022-03-23

**Authors:** Yan Huang, Xia Han, Ting Chang, Feng-fei Li, Xuan Chen, Yu-qing She

**Affiliations:** 1 Department of Endocrinology, The Fourth Affiliated Hospital of Nantong University, The First People’s Hospital of Yancheng, Nanjing, China; 2 Department of Endocrinology, Zhimaying Community Health Service Center, Qinhuai District, Nanjing, China; 3 Department of Nursing School, Nanjing University of Chinese Medicine, No. 282 Hanzhong Road, Nanjing, 210023 China; 4 Department of Endocrinology, Nanjing First Hospital, Nanjing Medical University, Nanjing, China; 5 Department of Endocrinology, Nanjing Pukou Central Hospital, Pukou Branch Hospital of Jiangsu Provence Hospital, No. 166 Shanghe Street, Pukou District, Nanjing, 210056 China

**Keywords:** Diseases, Endocrinology

## Abstract

Evidences indicate that elevated levels of circulating ErbB2 are closely associated with increased incidence of diabetes. However, the relationship between ErbB2 concentration and glycemic variations (GV) in type 2 diabetic (T2D) patients remains elucidated. The aim of this study was to assess whether there is an association between serum ErbB2 concentration and GV in newly diagnosed T2D patients. This was a three-center, and observational study. Between April 2019 and July 2019, a total of 106 newly diagnosed T2D patients were recruited. All recruited subjects were admitted as inpatients and received anti-diabetes agents free during the study period. At baseline, fasting serum was collected for ErbB2 measurement and all recruited patients were subjected a prospective CGM for at least 3 days. The primary endpoint was the relationships between ErbB2 concentrations and GV in T2D patients. Data of a total of 95 subjects who met the inclusion criteria were analyzed at the endpoint. Subjects were divided into quartiles according to their serum ErbB2 concentrations. We observed that subjects with an elevated level of ErbB2 had a higher value of GV in terms of mean amplitude of glucose excursion (MAGE), standard deviation of mean glucose (SDMG), and the coefficient of variation (CV%) than those with lower levels (all P < 0.05). Multiple linear regression analyzes after adjusting for confounder factors indicate that serum ErbB2 levels were significantly positively correlated with the MAGE (β = 0.664, t = 7.218, P < 0.01), SD (β = 0.469, t = 5.125, P < 0.01) and CV% (β = 0.337, t = 4.442, P < 0.01), respectively. Our data indicated that diabetic patients with higher ErbB2 concentrations may have large GV, which is an independent risk factor for microvascular and macrovascular complications.

## Introduction

The ErbB2/HER2 oncoprotein, a member of the receptor of tyrosine kinases, is a transmembrane protein, that consists of the internal tyrosine residues, the transmembrane portion, and the external extracellular domain (ECD)^1,2^. Homo- or heterodimerization leads to ErbB2 activation, which initiates a multiple of downstream signaling pathways that regulate cell proliferation, apoptosis, and differentiation^3^. Various studies have shown that serum ErbB2/HER2 concentration elevation is a specific marker for a variety of cancers, such as breast cancers^4,5^, ovarian, bladder, salivary gland, endometrial, pancreatic, and non-small-cell lung cancer^6^. Studies have also indicated that tumor patients with ErbB2 amplification or overexpression have an enhanced risk of poorer prognoses^7^.

Epidemiological studies have shown that diabetic patients have a potentially increased risk of increase in incidence of cancers^8^, and pre-diagnosed diabetic patients with tumors had an increased in all-cause and cancer-related mortality compared with individuals with normal glucose metabolism individuals^9,10^. The underlying biological mechanisms between the two heterogeneous, chronic and progressive diseases may be a partial reason for the two conditions to share the same metabolic risk factors, such as aging, obesity, and diet^8^. The links between diabetes and cancer are further strengthened by activation of the insulin receptor, insulin-like growth factor receptor (IGFR)^11^ and ErbB2^12^. Studies have demonstrated that ErbB2 plays a role in controlling lipid metabolism^13^ and is involved in impaired glucose metabolism^14^. This Fuels the idea that ErbB2 may be a bridge between diabetes and cancers, in that anti-diabetic agents may be a somewhat beneficial therapy for patients with cancer^15^. This hypothesis was confirmed by an in vitro study that found glucose-lowering agent metformin has an anti-cancer effect by mTOR/p70S6K1-sensed reactive oxygen species downregulating HER2 expression^12^.

Recently, a population-based cohort study confirmed that elevated levels of ErbB2 are associated with an increased incidence of diabetes^16^. Continued efforts have been made to suppress rapid glycemic variations in patients with type 2 diabetes (T2D)^17^. Large glucose fluctuations in patients with diabetes may have implications for the risk of long-term diabetic complications^18,19^. The underlying mechanisms might be acute glucose fluctuations, more specifically, the triggering of oxidative stress by acutely increased postprandial blood glucose levels^20^. However, the effects of serum ErbB2 concentrations on the glycemic variation in patients with T2D have not yet been elucidated.

Therefore, we performed a three-center, observational study using continuous glucose monitoring (CGM) to assess the relationship between ErbB2 concentrations and glucose variations in patients with T2D.

## Methods

This was a three-center, observational study. Between April 2019 and July 2019, 106 newly diagnosed patients with T2D were recruited from the following institutions in China: Department of Endocrinology, Yancheng No. 1 People’s Hospital, The Fourth Affiliated Hospital of Nantong University, Department of Endocrinology, Nanjing Pukou Central Hospital, Pukou Branch Hospital of Jiangsu Provence Hospital, and Zhimaying Community Health Service Center, Qinhuai District, Nanjing. The inclusion criteria were the following patients: (1) newly diagnosed patients with T2D random blood glucose less than 22.2 mmol/L; (2) HbA_1c_ < 12.0%; (3) aged between 18 and 80 years; and (4) body mass index (BMI) 21 to 35 kg/m^2^. The exclusion criteria were the following patients: (1) admission blood glucose higher than 22.2 mmol/L; (2) with chronic kidney or liver disease, and/or (3) diagnosed with maturity-onset diabetes in the young^21,22^. The study was approved by the ethics committee of Yancheng No. 1 People’s Hospital, The Fourth Affiliated Hospital of Nantong University, China. Written informed consent was obtained from all the patients. The methods, including all relevant details, were conducted in accordance with the Declaration of Helsinki guidelines.

All recruited patients were admitted as inpatients and were not treated with anti-diabetic agents during the study period. Oral glucose tolerance tests were performed at 8 a.m. the day after admission using 75 g glucose diluted in 100 mL water, and serum samples for the measurement of HbA_1c_, glucose, insulin, and C-peptide levels were obtained at 0, 30, and 120 min after glucose loading. Insulin concentration was measured using an insulin radioimmunoassay kit (Beijing Technology Company, Beijing, China). HbA_1c_ values were measured using a DiaSTAT HbA_1c_ analyzer (Bio-Rad, Hercules, CA, USA). C-peptide and glucose concentrations were measured centrally at the central laboratory of Yancheng No. 1 People’s Hospital. Fasting ErbB2 concentrations were measured using a commercially available quantitative enzyme-linked immunosorbent assay kit (Oncogene Science, Inc., Uniondale, NY, USA; Bayer AG, Leverkusen, Germany) according to the manufacturer’s instructions.

After collection of the baseline data, prospective CGM (Sof-sensor, CGMS-Gold, Medtronic plc, LA, CA, USA) was performed for 3 d, as described previously^23,24^. Briefly, the subcutaneous glucose sensor was embedded in the abdomen on day 1 at approximately 4 p.m. and was removed on day 4 at 4 p.m. During the CGM period, at least four finger-stick readings were entered for calibration every day. After the sensors were removed, CGM data were recorded by the investigators, as described previously^23–25^. The patients were instructed to maintain moderate activity while having breakfast, lunch, and dinner at 7 a.m., 11 a.m. and 5 p.m., respectively. Their meals consisted of carbohydrate, proteins, and fats in 55%, 17%, and 28% ratios, respectively.

The readings delivered from the CGM were recorded by the researchers. The 24-h mean glucose concentration (MG), the standard deviation of MG (SDMG), the coefficient of variation (CV%), the incremental area under the curve (AUC) of glucose concentrations above 10.0 mmol/L or the incremental area over the curve (AOC) less than 3.9 mmol/L, and the time in range (TIR) of glucose levels ranged from 3.9 to 10.0 mmol/L, and the hourly MGs were calculated by researchers. The mean amplitude of glucose excursion (MAGE) was calculated manually, as previously described^23,24^. In addition, β-cell function and insulin sensitivity were assessed using the homoeostasis model assessment B (HOMA-B), HOMA-insulin resistance (IR)^21,26^ and the Matsuda index^27,28^.

The primary endpoint was the relationship between ErbB2 concentrations and glucose variations in patients with T2D. The differences in 24-h MG, SDMG, incremental AUC (glucose > 10.0 mmol/L), incremental AOC (glucose < 3.9 mmol/L), TIR, hourly MG, β-cell function, and insulin sensitivity in patients with different ErbB2 concentrations were also analyzed.

### Statistical analysis

The normal distribution data were presented as mean ± SD. Statistical analysis was performed using the SPSS software (version 17.0; IBM Corp., Armonk, NY, USA). The Shapiro–Wilk test was used to verify data distribution. A test was performed to compare the ratio differences between the two groups. All of the repeated data were analyzed via a two-way analysis of variance between groups, followed by the Bonferroni–Dunn post hoc test. P-values were two-tailed, with a significance level of 5%. Multiple linear regression analyses were performed to assess the correlation between ErbB2 and MAGE.

### Ethics approval and consent to participate

The study was approved by the ethics committee of Yancheng No. 1 People’s Hospital, The Fourth Affiliated Hospital of Nantong University, China. All patients gave written informed consent. The methods were conducted in accordance with the Declaration of Helsinki guidelines, including any relevant details.

### Consent for publication

Written informed consent for publication was obtained from all participants.

## Results

### Baseline characteristics

A total of 106 patients newly diagnosed with T2D between April 2019 and July 2019 were screened for eligibility at the following institutions in China: Department of Endocrinology, Yancheng No. 1 People’s Hospital, The Fourth Affiliated Hospital of Nantong University; Department of Endocrinology, Nanjing Pukou Central Hospital, Pukou Branch Hospital of Jiangsu Provence Hospital; and Zhimaying Community Health Service Center, Qinhuai District, Nanjing. Eleven patients were excluded from the analysis, five due to having capillary glucose concentrations above 22.2 mmol/L and six due to having readings of CGM missing for at least 10%. The remaining 95 patients who met the inclusion criteria (53 men and 42 women, aged 55.1 ± 7.6 years, BMI 24.7 ± 3.6 kg/m^2^, and HbA1c values 9.2 ± 1.8%) were analyzed at the endpoint. We observed that patients with newly diagnosed T2D had ErbB2 concentrations ranging from 13.2 ± 3.8 to 6.3 ± 2.2 ng/ml.

### Glycemic variations in subjects with different ErbB2 concentrations

We analyzed data from day 1 to day 3 of CGM at the endpoint because CGM has an infiltration phage at the beginning of the monitoring period and a sensor expires phage at the end of the monitoring period, which might not be reliable according to the manufacturer’s instructions. The included patients were subjected to CGM at 1600–1500, yielding 751 ± 76 glucose readings per patient.

To observe whether patients with various ErbB2 concentrations had the same glycemic variations, the patients were assigned into quartiles according to their serum ErbB2 concentrations. Demographic data were comparable across the four groups, as shown in Table 1. The differences in glycemic variation are presented in Table 2. We observed that patients with an elevated level of ErbB2 generally had a higher value of GV in terms of MAGE, SD, and CV% (all P < 0.05) than did those with lower levels (Table 2).

**Table 1:** Baseline characteristics of subjects across 4 quartiles of ErbB2 concentrations.

Items	Quartile 1	Quartile 2	Quartile 3	Quartile 4	P value
Number	23	24	24	24	–
Male	11	15	16	11	0.66
Age	54.6 ± 10.8	52.1 ± 12.5	56.7 ± 8.9	53.2 ± 12.0	0.78
BMI	23.3 ± 5.6	24.1 ± 6.0	25.0 ± 8.4	23.9 ± 7.2	0.81
HbA1c	9.0 ± 2.0	9.5 ± 4.6	8.8 ± 1.7	9.3 ± 2.5	0.82
Waistline	78.1 ± 28.4	74.6 ± 20.1	82.3 ± 19.6	75.8 ± 23.0	0.79
Waist-to-hip	0.8 ± 0.4	0.8 ± 0.2	0.8 ± 0.3	0.8 ± 0.2	0.92
Triglycerides	1.2 ± 1.3	1.4 ± 1.0	1.4 ± 1.5	1.8 ± 1.3	0.04*
Cholesterol	4.6 ± 1.5	5.4 ± 1.2	5.3 ± 1.8	5.8 ± 1.1	0.03*

Data were presented as means ± SD. *Age* (years); *BMI* body mass index (kg/m^2^), *HbA1c* glycated hemoglobin (%), Waistline (cm), *Waist-to-hip* waist-to-hip ratio (%), Triglyceride (mmol/L), Cholesterol (mmol/L). *P < 0.05 (both Quartile 1 vs. Quartile 4).

**Table 2:** Glycemic profiles in recruited subjects across 4 quartiles of ErbB2 concentrations.

Items	Quartile 1	Quartile 2	Quartile 3	Quartile 4	P value
Number	23	24	24	24	–
24-h MG	8.7 ± 1.9	8.6 ± 3.2	8.3 ± 1.2	8.2 ± 2.1	0.18
SDMG	1.6 ± 1.1	1.9 ± 1.8	2.2 ± 1.0	2.6 ± 1.3	0.03
MAGE	5.4 ± 2.1	5.7 ± 1.2	6.4 ± 1.0	7.1 ± 2.6	0.01
CV%	18.4 ± 12.3	22.7 ± 20.5	26.7 ± 21.6	31.7 ± 21.6	0.02
TIR	72.1 ± 28.0	64.3 ± 21.1	42.9 ± 31.4	35.6 ± 23.2	0.00
AUC > 10.0	0.3 ± 0.4	0.5 ± 0.2	0.6 ± 0.3	0.8 ± 0.2	0.00
AUC < 3.9	0.3 ± 0.0	0.2 ± 0.0	0.0 ± 0.0	0.0 ± 0.0	0.23

Data were presented as means ± SD, *P < 0.05. *24-h MG* 24-h mean glucose concentrations (mmol/L), *SDMG* 24-h standard deviation of mean glucose concentrations (mmol/L), *MAGE* 24-h mean amplitude of glycemic excursions (mmol/L), *CV%* the coefficient of variation (%), *TIR* time in range from > 3.9 mmol/L to < 10.0 mmol/L (%), *AUC* > 10.0 *mmol/L* the incremental AUC of glucose level above 10.0 mmol/L (mmol/L*Day), *AUC* < *3.9 mmol/L* the incremental AUC of glucose less than 3.9 mmol/L (mmol/L*Day).

In the present study, our CGM data showed that the 24-h SDMG, MAGE, CV%, and the incremental AUC of glucose above 10 mmol/L were progressively and significantly amplified alongside ErbB2 levels in patients newly diagnosed with T2D. As expected, patients in the higher quartiles of ErbB2 levels were significantly fewer in the TIR group than in the lower quartiles (Table 2).

In addition, we did not observe statistically significant differences in the 24-h MG and incremental AOC of hypoglycemia across the quartiles in the recruited patients. In addition, no hypoglycemic episode (defined as finger-stick glucose < 3.9 mmol/L and/or symptomatic hypoglycemia) was reported during the study period. However, our CGM data demonstrated that six patients in the highest quartile and one patient in the third quartile experienced hypoglycemic episodes (defined as CGM glucose reading < 3.9 mmol/L), with hypoglycemic durations ranging from 10 to 60 min. We did not observe hypoglycemic episodes delivered by CGM in patients in the other two quartiles.

### Relationships between ErbB2 concentration and glycemic variations

Multiple linear regression analyzes were performed to assess the correlation between ErbB2 and MAGE. Our data indicated that HbA1c, age, BMI, ErbB2, FBG, MG, HOMA-IR, SDMG, sex, smoking habits, C-reactive protein (CRP), systolic blood pressure, and low-density lipoprotein (LDL) cholesterol remained significant in the stepwise regression analysis. The standardized regression coefficients were 0.516 (t = 6.27, P < 0.01), 0.496 (t = 6.112, P < 0.01), 0.464 (t = 5.634, P < 0.01), 0.433 (t = 5.535, P < 0.01), 0.283 (t = 3.218, P < 0.01), − 0.201 (t = − 3.482, P < 0.05) and 0.175 (t = 2.387, P < 0.05), respectively. After controlling for HbA_1c_, age, BMI, ErbB2, FBG, MG, HOMA-IR, SDMG, sex, smoking habits, CRP, systolic blood pressure, and LDL cholesterol, the ErbB2 concentration was still significantly positively correlated with MAGE 0.664 (t = 7.218, P < 0.01). Similarly, ErbB2 concentration was significantly positively correlated with SD (β = 0.469, t = 5.125, P < 0.01) and CV% (β = 0.337, t = 4.442, P < 0.01).

### Relationships between ErbB2 concentration and β-cell function/insulin sensitivity

We also observed the relationship between ErbB2 concentration and β-cell function/insulin sensitivity in patients with different ErbB2 levels. Our data showed that patients with higher ErbB2 concentrations exhibited an increase in HOMA-IR values and induction of the Matsuda index (Table 3). However, there were no differences in the HOMA-B values across the quartiles of ErbB2 concentrations. Multivariate analysis controlled for age and BMI to determine the relationships between ErbB2 concentration and β-cell function and insulin sensitivity. Our data showed that ErbB2 values were significantly negatively correlated with HOMA-IR (β = 0.422, t = 4.117, P < 0.01) and Matsuda index (β = 0.317, t = 2.885, P < 0.05). We did not observe a statistically significant relationship between ErbB2 concentration and β-cell function.

**Table 3:** The β-cell function and insulin sensitivity in recruited subjects across 4 quartiles of ErbB2 concentrations.

Items	Quartile 1	Quartile 2	Quartile 3	Quartile 4	P value
Number	23	24	24	24	–
C-peptide 0	2.8 ± 0.1	2.7 ± 1.3	2.2 ± 0.3	2.2 ± 1.1	0.23
C-peptide 30	3.8 ± 1.7	3.2 ± 0.5	2.9 ± 3.2	2.8 ± 2.7	0.42
C-peptide 120	5.2 ± 2.3	5.4 ± 0.8	5.4 ± 1.1	5.1 ± 2.7	0.36
HOMA-B	19.1 ± 15.4	20.3 ± 21.0	21.7 ± 17.2	18.9 ± 16.6	0.66
HOMA-IR	2.1 ± 2.3	2.6 ± 1.1	3.4 ± 1.0	3.8 ± 1.1	0.04*
Mastuda Index	116.8 ± 81.5	107.1 ± 22.7	96.3 ± 12.8	85.0 ± 33.9	0.03*

Data were presented as means ± SD, *P < 0.05. *C-peptide 0* C-peptide 0 min (ng/mL), *C-peptide 30* C-peptide 30 min (ng/mL), *C-peptide 120* C-peptide 120 min (ng/mL), *HOMA-B* the homoeostasis model assessment B, *HOMA-IR* the homoeostasis model assessment IR.

### Relationships between ErbB2 concentration and lipid profiles

Furthermore, because ErbB2 control lipid metabolism^13^. we assess the relationship between ErbB2 concentration and lipid profiles in patients with different ErbB2 levels. We found that the patients in the highest ErbB2 concentration group had statistically significant increases in triglycerides and cholesterol values than did those in the lowest ErbB2 concentration group (both P < 0.05).

## Discussion

In this observational study, we found that patients with newly diagnosed T2D with higher serum ErbB2 concentrations exhibited increased glycemic variations with respect to MAGE, SD, and CV% than did the patients with lower serum ErbB2 concentrations. Multivariate linear regression analyses of the whole study population revealed that serum ErbB2 concentration was positively correlated with glycemic variations, and this association remained significant after adjusting for potential confounding factors, such as age, sex, and BMI. Our data also showed that patients with T2D with higher serum ErbB2 concentrations had an increase in IR in terms of the HOMA-IR and Matsuda index than did those with lower ErbB2 concentrations.

It was well-documented that ErbB2 is been widely investigated as an oncogenic marker and the predictor of cancer prognosis^7^. There is a close association between metabolism and IR, which prompts the confirmation of a possible link between ErbB2 and glucose metabolism in future studies. Recent in vivo and in vitro studies have observed a role beyond the oncogenesis of ErbB2, with regard to the relationship between ErbB2 and diabetes^15^. Fatty acid synthase (FASN) activity may partly account for the link between ErbB2 and diabetes because the overexpression of ErbB2 through a phosphatidylinositol 3'-kinase-dependent pathway promotes the expression of FASN^29^. Studies have provided ample evidence indicating that circulating FASN is a candidate biomarker for the diagnosis and prognosis for diabetes^30^, and it has been associated with insulin action, glucose metabolism, and resistance in the development of T2D^31^. A study performed by Fernandez-Real et al.^32^ found that serum ErbB2 concentrations were positively associated with IR in obese persons, indicating that ErbB2 might play a role in the pathophysiology of diabetes^32^. Furthermore, other studies have provided strong evidence supporting the notion that higher serum ErbB2 levels may rely on ErbB2, leading to an increase in IR^32^, and hyperglycemia^32,33^. As with previous studies, our data showed that patients with T2D higher serum ErbB2 concentrations had an increase in IR in terms of HOMA-IR and Matsuda index compared to those with lower ErbB2 concentrations. However, we did not observe any difference in HOMA-B across the quartiles in any of the recruited patients.

A significant increase in serum ErbB2 concentrations along with the deterioration of glucose metabolism was observed^33^. Ashfaque et al. found that serum ErbB2 levels were significantly higher in patients with T2D than in those with impaired fasting glucose (IFG) and/or impaired glucose tolerance (IGT), and those with IFG and IGT had a higher ErbB2 compared to those with normal glucose tolerance, after adjusting for age, sex, and body mass index^33^. In the present study, we did not have data regarding the differences in ErbB2 concentrations in populations with different glucose metabolism conditions. However, we observed that diabetic patients with higher ErbB2 levels had larger GV than did those with lower serum ErbB2 concentrations. Microvascular and macrovascular complications are mainly^34,35^, or at least partially^35,36^, dependent on hyperglycemia. Postprandial glucose increase is a well-know independent risk factor for cardiovascular disease^37^, as is chronic hyperglycemia, which may trigger oxidative stress^20^, and induce an overproduction of peroxynitrite and nitrotyrosine in patients with T2D^20,38,39^.

We also analyzed the relationship between serum ErbB2 concentrations and GV, β-cell function, and IR. As with a previous study^32,33^, our multivariate linear analysis after adjusting for the confounding factors, patients with higher ErbB2 values had higher HOMA-IR values and decreased Matsuda index values. We did not observe a correlation between ErbB2 values and β cell function in the present study. Notably, our data showed that serum ErbB2 concentrations were strongly positively correlated with GV (MAGE, SD, and CV%). However, we do not have data to support these observation, and future studies are needed to ascertain the underlying mechanisms of ErbB2 that lead to amplified glycemic fluctuations.

Diabetic patients must achieve HbA_1c_ target values before physicians can prescribe glucose-lowering agents^40^. However, HbA_1c_ does not provide sufficient evidence of daily glucose variations^18,19^. Studies have demonstrated that glucose variations, especially large MAGE, are independent risk factors for long-term diabetic complications in T2D^18,19^. The underlying mechanisms may be the reason by acute glucose variations triggering multiple factors, that impair the epithelial cells^20^.

This study had certain limitations. First, a correlation between HbA1c and glycemic variation has been reported in previous studies^22^. In agreement with our findings, it would be more logical that patients with higher ErbB2 values exhibited higher HbA1c concentrations. However, there was no positive correlation between ErbB2 levels and HbA1c concentrations. We speculated that the following two reasons may have impaired this correlation: (1) our recruited patients had moderate HbA1c concentrations, ranging from 8.1 to 14.1%, with only six patients exhibiting an HbA1c concentration higher than 10%; (2) the sample size was moderate, which may have been the primary reason for the decrease in the possibility of the correlation. Second, we did not observe a significant difference in serum ErbB2 concentration between non-diabetic and diabetic patients.

In conclusion, our data indicated that diabetic patients with higher ErbB2 concentrations may have large GV, which is an independent risk factor for microvascular and macrovascular complications.

## Data Availability

The data sets generated during and/or analyzed during the current study are not publicly available but are available from the corresponding author on reasonable request.

## References

[1] Rubin I, Yarden Y (2001). The basic biology of HER2. Ann. Oncol..

[2] Molina R, Escudero JM, Munoz M, Auge JM, Filella X (2012). Circulating levels of HER-2/neu oncoprotein in breast cancer. Clin. Chem. Lab. Med..

[3] Iqbal N (2014). Human epidermal growth factor receptor 2 (HER2) in cancers: Overexpression and therapeutic implications. Mol. Biol. Int..

[4] Burstein HJ (2005). The distinctive nature of HER2-positive breast cancers. N. Engl. J. Med..

[5] Yan M, Parker BA, Schwab R, Kurzrock R (2014). HER2 aberrations in cancer: Implications for therapy. Cancer Treat. Rev..

[6] Scholl S, Beuzeboc P, Pouillart P (2001). Targeting HER2 in other tumor types. Ann. Oncol..

[7] Menard S, Fortis S, Castiglioni F, Agresti R, Balsari A (2001). HER2 as a prognostic factor in breast cancer. Oncology.

[8] Giovannucci E (2010). Diabetes and cancer: A consensus report. CA Cancer J. Clin..

[9] Barone BB (2008). Long-term all-cause mortality in cancer patients with preexisting diabetes mellitus: A systematic review and meta-analysis. JAMA.

[10] Rao Kondapally Seshasai S (2011). Diabetes mellitus, fasting glucose, and risk of cause-specific death. N. Engl. J. Med..

[11] Frasca F (2008). The role of insulin receptors and IGF-I receptors in cancer and other diseases. Arch. Physiol. Biochem..

[12] Vazquez-Martin A, Oliveras-Ferraros C, Menendez JA (2009). The antidiabetic drug metformin suppresses HER2 (erbB-2) oncoprotein overexpression via inhibition of the mTOR effector p70S6K1 in human breast carcinoma cells. Cell Cycle.

[13] Ray A (2017). Tumor-linked HER2 expression: Association with obesity and lipid-related microenvironment. Horm. Mol. Biol. Clin. Investig..

[14] Ferroni P (2015). Type 2 diabetes and breast cancer: The interplay between impaired glucose metabolism and oxidant stress. Oxid. Med. Cell. Longev..

[15] Jalving M (2010). Metformin: Taking away the candy for cancer?. Eur. J. Cancer.

[16] Muhammad IF (2019). Circulating HER2/ErbB2 levels are associated with increased incidence of diabetes: A population-based cohort study. Diabetes Care.

[17] Gallwitz B (2009). Implications of postprandial glucose and weight control in people with type 2 diabetes: Understanding and implementing the International Diabetes Federation guidelines. Diabetes Care.

[18] Nathan DM (2008). Translating the A1C assay into estimated average glucose values. Diabetes Care.

[19] Del Prato S (2002). In search of normoglycaemia in diabetes: Controlling postprandial glucose. Int. J. Obes. Relat. Metab. Disord..

[20] Monnier L (2006). Activation of oxidative stress by acute glucose fluctuations compared with sustained chronic hyperglycemia in patients with type 2 diabetes. JAMA.

[21] Weng J (2008). Effect of intensive insulin therapy on beta-cell function and glycaemic control in patients with newly diagnosed type 2 diabetes: A multicentre randomised parallel-group trial. Lancet.

[22] Li FF (2017). Features of glycemic variations in drug naive type 2 diabetic patients with different HbA1c values. Sci. Rep..

[23] Li FF (2015). Influence of acarbose on plasma glucose fluctuations in insulin-treated patients with type 2 diabetes: A pilot study. Int. J. Endocrinol..

[24] Li FF (2016). Blood glucose fluctuations in type 2 diabetes patients treated with multiple daily injections. J. Diabetes Res..

[25] Zhou J (2009). Reference values for continuous glucose monitoring in Chinese subjects. Diabetes Care.

[26] Matthews DR (1985). Homeostasis model assessment: Insulin resistance and beta-cell function from fasting plasma glucose and insulin concentrations in man. Diabetologia.

[27] Matsuda M, DeFronzo RA (1999). Insulin sensitivity indices obtained from oral glucose tolerance testing: Comparison with the euglycemic insulin clamp. Diabetes Care.

[28] Sasaki R (2016). Association of waist circumference and body fat weight with insulin resistance in male subjects with normal body mass index and normal glucose tolerance. Intern. Med..

[29] Kumar-Sinha C, Ignatoski KW, Lippman ME, Ethier SP, Chinnaiyan AM (2003). Transcriptome analysis of HER2 reveals a molecular connection to fatty acid synthesis. Cancer Res..

[30] Fernandez-Real JM (2010). Extracellular fatty acid synthase: A possible surrogate biomarker of insulin resistance. Diabetes.

[31] Menendez JA, Vazquez-Martin A, Ortega FJ, Fernandez-Real JM (2009). Fatty acid synthase: Association with insulin resistance, type 2 diabetes, and cancer. Clin. Chem..

[32] Fernandez-Real JM (2010). Serum HER-2 concentration is associated with insulin resistance and decreases after weight loss. Nutr. Metab..

[33] Memon AA (2015). Circulating human epidermal growth factor receptor 2 (HER2) is associated with hyperglycaemia and insulin resistance. J. Diabetes.

[34] Diabetes Control and Complications Trial Research Group (1995). The relationship of glycemic exposure (HbA1c) to the risk of development and progression of retinopathy in the diabetes control and complications trial. Diabetes.

[35] Klein R (1995). Hyperglycemia and microvascular and macrovascular disease in diabetes. Diabetes Care.

[36] Stratton IM (2000). Association of glycaemia with macrovascular and microvascular complications of type 2 diabetes (UKPDS 35): Prospective observational study. BMJ.

[37] Nakagami T (2004). Hyperglycaemia and mortality from all causes and from cardiovascular disease in five populations of Asian origin. Diabetologia.

[38] Ceriello A (2008). Oscillating glucose is more deleterious to endothelial function and oxidative stress than mean glucose in normal and type 2 diabetic patients. Diabetes.

[39] Hu Y, Liu W, Huang R, Zhang X (2010). Postchallenge plasma glucose excursions, carotid intima-media thickness, and risk factors for atherosclerosis in Chinese population with type 2 diabetes. Atherosclerosis.

[40] Inzucchi SE (2015). Management of hyperglycemia in type 2 diabetes, 2015: A patient-centered approach: Update to a position statement of the American Diabetes Association and the European Association for the Study of Diabetes. Diabetes Care.

